# Supplementary material for: Mass-Rearing Conditions Do Not Always Reduce Genetic Diversity: The Case of the Mexican Fruit Fly, Anastrepha ludens (Diptera: Tephritidae)
Source: Insects. 2024 Jan 12;15(1):56. doi: 10.3390/insects15010056 (PMC10816967; doi:10.3390/insects15010056)
Supplement: Supplementary file 1 [file insects-15-00056-s001.zip › insects-2757247-supplementary.pdf]

Table S1. Original data set of genotypes with nuclear DNA fragments of seven microsatellites of samples *Anastrapheba ludens* Loew from field and mass-reared. LAB = laboratory strain; TA7 = Tapachula-7 strain; WIL = wild strain. Each line corresponds to an individual, and by column are given its diploid genotype per microsatellite.

**Pop**	**Microsatellite**													
	Asus1-1H		Asus1-2B		Asus1-2F		Asus-3B		Asus1-4H		Asus1-6C		Asus1-8D	
LAB	225	225	135	135	115	125	105	105	185	185	495	505	205	225
LAB	215	215	115	125	285	295	105	105	185	185	495	495	265	265
LAB	215	215	115	115	275	305	105	105	185	185	505	505	215	215
LAB	215	215	115	115	295	295	105	105	185	185	505	505	205	205
LAB	215	215	115	125	295	295	125	135	185	185	505	505	215	215
LAB	215	215	125	125	295	295	105	105	155	155	495	505	215	255
LAB	205	205	115	115	295	295	105	105	185	195	505	505	205	205
LAB	215	215	115	115	295	295	105	105	245	245	495	495	215	215
LAB	215	215	125	135	275	295	105	105	185	185	485	495	205	205
LAB	215	215	135	135	285	295	105	105	185	185	485	495	205	205
LAB	215	215	115	115	275	295	105	105	295	295	485	505	205	205
LAB	215	215	115	115	275	295	105	105	185	185	485	495	205	205
LAB	215	215	115	115	295	295	105	105	185	185	485	505	205	205
LAB	215	215	115	115	295	295	105	105	185	185	485	495	-1	-1
LAB	215	215	115	115	275	305	115	125	185	185	505	505	-1	-1
LAB	215	215	115	115	295	295	105	105	155	155	485	495	215	215
LAB	215	215	175	215	295	295	105	105	155	155	495	495	215	215
LAB	215	215	115	115	275	295	105	105	155	155	495	495	215	215
LAB	215	215	115	125	295	295	105	105	185	185	505	505	215	215
LAB	215	215	115	115	295	295	105	105	185	185	495	495	215	215
LAB	215	215	125	125	275	295	105	105	185	185	485	495	205	205
LAB	215	215	115	125	275	295	105	105	185	185	485	505	205	205
LAB	215	215	115	125	295	295	105	105	185	185	485	505	205	205
LAB	215	215	125	125	275	295	105	105	185	185	485	505	-1	-1
LAB	215	215	115	115	295	295	105	105	185	185	485	495	205	205
LAB	215	215	115	115	295	295	105	105	185	185	485	495	205	205
LAB	215	215	115	115	275	305	105	105	185	185	485	495	205	205
LAB	215	215	115	125	305	305	105	105	185	185	485	505	215	215
LAB	215	215	125	135	295	295	-1	-1	155	155	505	505	215	215
LAB	215	215	115	125	295	295	-1	-1	185	185	495	495	-1	-1
TA7	215	215	115	115	275	275	115	115	185	185	485	495	205	215
TA7	215	215	125	125	275	275	105	105	195	195	495	495	205	215
TA7	215	215	115	115	275	295	105	105	185	185	485	495	265	265

TA7	215	215	125	135	275	275	105	105	185	185	485	495	265	265
TA7	215	215	125	125	275	295	105	105	185	185	485	505	275	295
TA7	215	215	115	115	-1	-1	105	105	195	195	505	505	275	275
TA7	215	215	115	115	-1	-1	105	105	195	195	495	505	265	265
TA7	215	215	135	145	-1	-1	105	105	195	195	495	595	265	265
TA7	215	215	115	115	305	305	105	105	195	195	495	495	265	265
TA7	215	215	115	115	305	305	105	105	185	195	495	495	265	295
TA7	215	215	115	115	275	275	105	105	185	185	485	505	265	265
TA7	215	215	135	145	275	295	105	105	195	195	495	495	205	215
TA7	215	215	115	115	275	275	105	105	195	195	485	495	205	205
TA7	215	215	135	135	275	295	105	105	195	195	485	505	265	265
TA7	215	215	115	115	275	295	105	105	195	195	485	495	265	265
TA7	215	215	115	115	-1	-1	105	105	185	185	495	505	265	265
TA7	215	215	125	135	-1	-1	105	105	185	185	485	505	275	275
TA7	215	215	125	135	-1	-1	105	105	185	185	485	505	275	275
TA7	215	215	125	125	-1	-1	105	105	195	195	465	495	265	265
TA7	215	215	135	135	-1	-1	105	105	185	195	465	495	265	265
TA7	215	215	135	135	275	275	105	105	195	195	485	495	265	265
TA7	215	215	115	115	275	275	105	105	195	195	465	485	265	265
TA7	175	215	135	135	275	275	105	105	195	195	485	495	265	265
TA7	215	215	125	135	275	275	105	105	195	195	485	495	255	265
TA7	175	215	135	135	275	295	105	105	195	195	465	485	265	285
TA7	215	215	125	125	-1	-1	105	105	185	185	485	495	215	215
TA7	175	215	115	115	295	295	105	105	185	185	465	485	265	265
TA7	175	215	115	115	-1	-1	105	105	195	195	485	495	275	275
TA7	115	115	115	125	295	295	105	105	185	195	475	485	275	275
TA7	175	215	125	135	295	295	115	115	185	195	505	505	275	275
TA7	215	215	125	125	285	295	105	105	195	195	495	495	265	265
WIL	215	215	115	115	275	295	115	115	185	195	485	495	205	205
WIL	215	215	115	125	275	305	105	105	185	195	485	495	205	205
WIL	215	215	115	115	295	295	105	105	185	195	495	495	205	205
WIL	215	215	195	195	295	295	105	105	195	195	505	505	205	205
WIL	215	215	115	115	295	295	105	105	195	195	505	505	205	205
WIL	215	215	115	125	295	295	105	105	195	195	505	505	265	265
WIL	215	215	115	135	275	275	105	105	185	195	485	495	205	205
WIL	215	215	115	125	275	275	105	105	195	195	485	495	205	205
WIL	215	215	125	135	275	295	105	115	195	195	485	495	205	205
WIL	215	215	115	125	295	295	105	105	185	185	485	495	215	215
WIL	215	215	115	115	285	295	105	105	185	195	475	485	215	215

WIL	215	215	115	115	295	295	105	105	195	195	495	495	205	205
WIL	215	215	115	115	275	295	105	105	185	185	485	495	205	205
WIL	215	215	115	125	275	275	105	105	185	185	495	495	205	205
WIL	215	215	115	125	275	275	105	105	185	185	485	495	265	265
WIL	215	215	115	115	295	295	105	105	195	195	485	495	265	265
WIL	215	215	115	115	295	295	105	105	195	195	485	495	205	205
WIL	215	215	115	115	295	295	105	105	195	195	485	495	205	205
WIL	215	215	115	115	295	295	-1	-1	195	195	485	505	205	205
WIL	215	215	115	115	295	295	115	115	185	185	465	485	205	205
WIL	215	215	115	115	275	275	-1	-1	185	185	495	505	-1	-1
WIL	215	215	115	115	-1	-1	-1	-1	155	155	535	545	215	215
WIL	215	215	135	145	275	275	-1	-1	185	185	485	505	215	215
WIL	215	215	115	115	155	155	105	115	195	195	595	595	215	215
WIL	215	215	115	115	295	295	95	95	195	195	485	495	195	195
WIL	215	215	115	115	-1	-1	105	105	195	195	485	495	205	215
WIL	215	215	115	115	305	305	105	115	185	185	485	495	-1	-1
WIL	215	215	115	125	275	295	105	105	195	195	485	495	205	205
WIL	215	215	115	135	275	275	105	105	195	195	465	485	-1	-1
